# Supplementary material for: Three-Step Synthesis of the Antiepileptic Drug Candidate Pynegabine
Source: Molecules. 2023 Jun 21;28(13):4888. doi: 10.3390/molecules28134888 (PMC10343641; doi:10.3390/molecules28134888)
Supplement: Supplementary file 1 [file molecules-28-04888-s001.zip › Supplementary Materials (1).pdf]

Three-step Synthesis of the Antiepileptic Drug Candidate Pynegabine

Yi-Jing Sun [1,2], Ya-Ling Gong [1], Shi-Chao Lu [1], Shi-Peng Zhang [1,]* and Shu Xu [1,2,]*

[1] State Key Laboratory of Bioactive Substance and Function of Natural Medicines and Beijing Key Laboratory of Active Substance Discovery and Druggability Evaluation, Institute of Materia Medica, Chinese Academy of Medical Sciences and Peking Union Medical College, 2A Nanwei Road, Xicheng District, Beijing 100050, China

[2] College of Chemical and Pharmaceutical Engineering, Hebei University of Science and Technology, Shijiazhuang 050018, Hebei, China

Table of Contents

1.	^{1}H NMR spectrum of compound **17** ($CDCl_3$, 400 MHz)	S2
2.	^{13}C NMR spectrum of compound **17** ($CDCl_3$, 101 MHz)	S3
3.	^{1}H NMR spectrum of compound **15** ($CDCl_3$, 400 MHz)	S4
4.	^{13}C NMR spectrum of compound **15** ($CDCl_3$, 101 MHz)	S5
5.	^{1}H NMR spectrum of compound **15** (d_6-DMSO, 400 MHz)	S6
6.	^{13}C NMR spectrum of compound **15** (d_6-DMSO, 101 MHz)	S7
7.	^{1}H NMR spectrum of pynegabine ($CDCl_3$, 400 MHz)	S8
8.	^{13}C NMR spectrum of pynegabine ($CDCl_3$, 101 MHz)	S9
9.	^{1}H NMR spectrum of pynegabine (d_6-DMSO, 400 MHz)	S10
10.	^{13}C NMR spectrum of pynegabine (d_6-DMSO, 101 MHz)	S11
11.	Elemental analysis results of pynegabine	S12

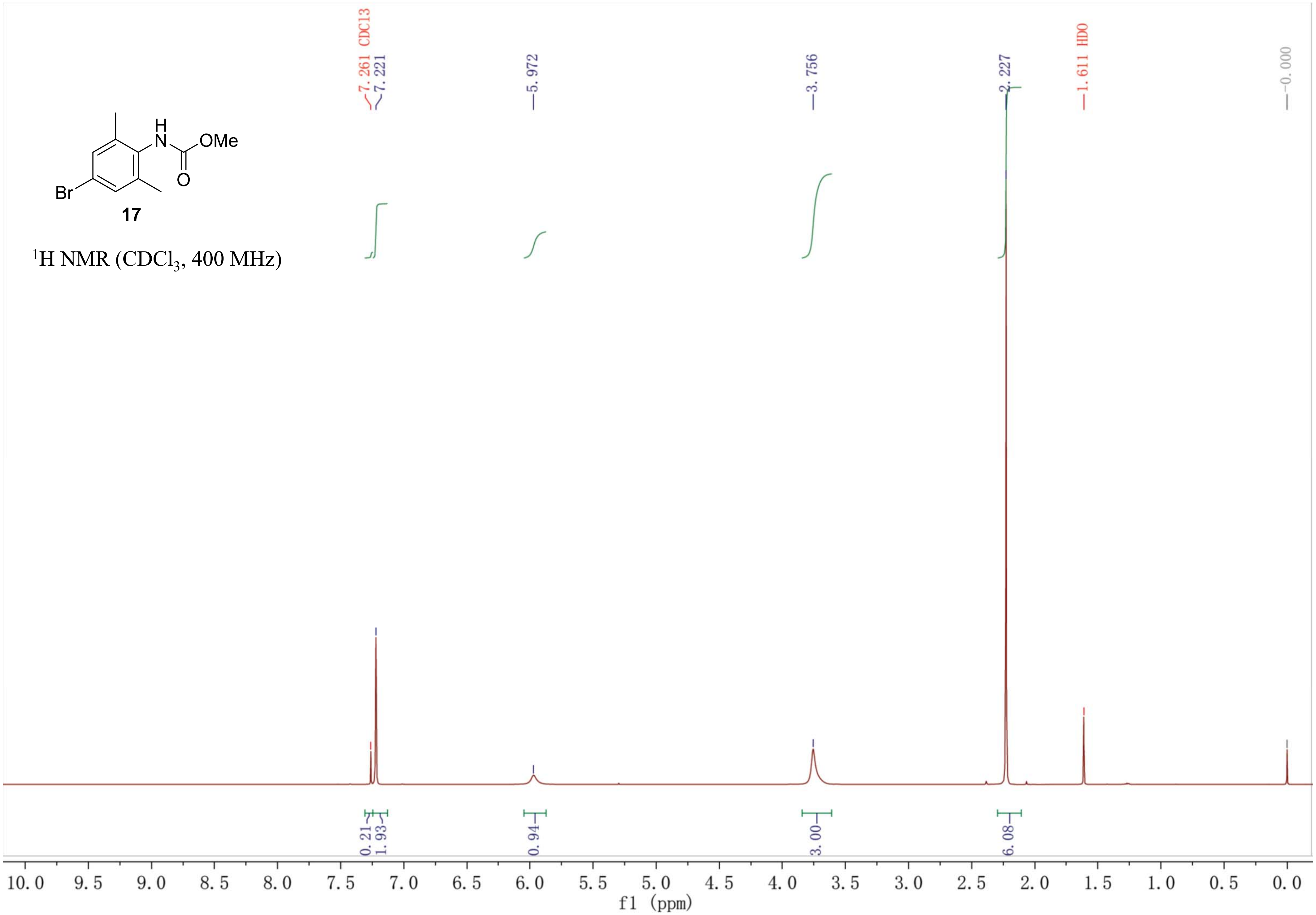
7.261 CDCl3
7.221
5.972
3.756
2.227
1.611 HDO
0.000
OMe
H
N
O
Br
17
^{1}H NMR ($CDCl_3$, 400 MHz)
0.21
1.93
0.94
3.00
6.08
10.0 9.5 9.0 8.5 8.0 7.5 7.0 6.5 6.0 5.5 5.0 4.5 4.0 3.5 3.0 2.5 2.0 1.5 1.0 0.5 0.0
f1 (ppm)

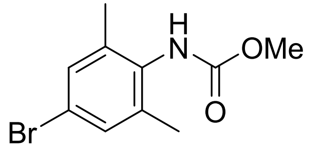

17

^{13}C NMR ($CDCl_3$, 101 MHz)

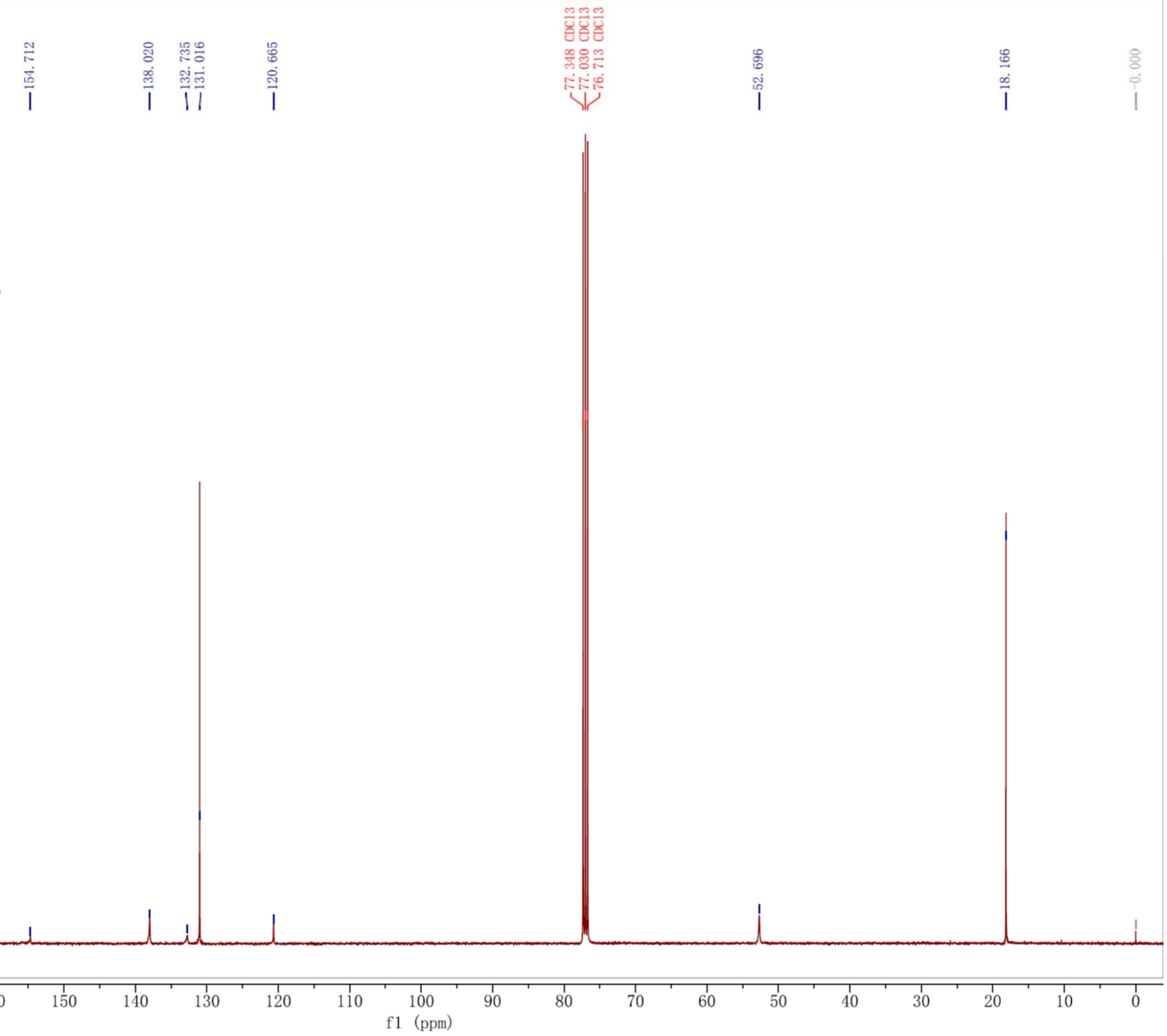

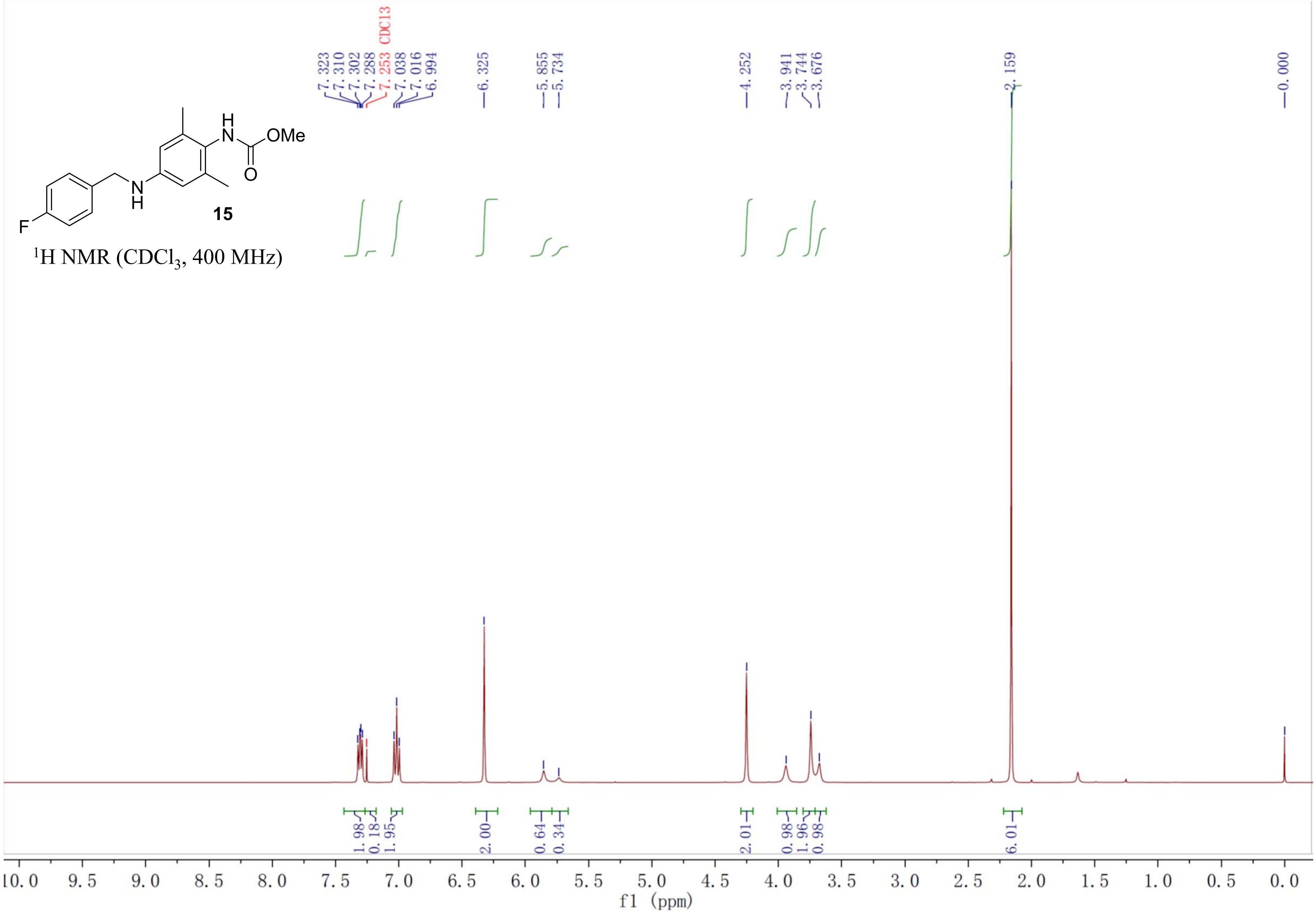

7.323
7.310
7.302
7.288
7.253 CDCl3
7.038
7.016
6.994
6.325
5.855
5.734
4.252
3.941
3.744
3.676
2.159
0.000
H
N
OMe
O
N
H
F
15
¹H NMR (CDCl3, 400 MHz)
1.98
0.18
1.95
2.00
0.64
0.34
2.01
0.98
1.96
0.98
6.01
10.0
9.5
9.0
8.5
8.0
7.5
7.0
6.5
6.0
5.5
5.0
4.5
4.0
3.5
3.0
2.5
2.0
1.5
1.0
0.5
0.0
f1 (ppm)

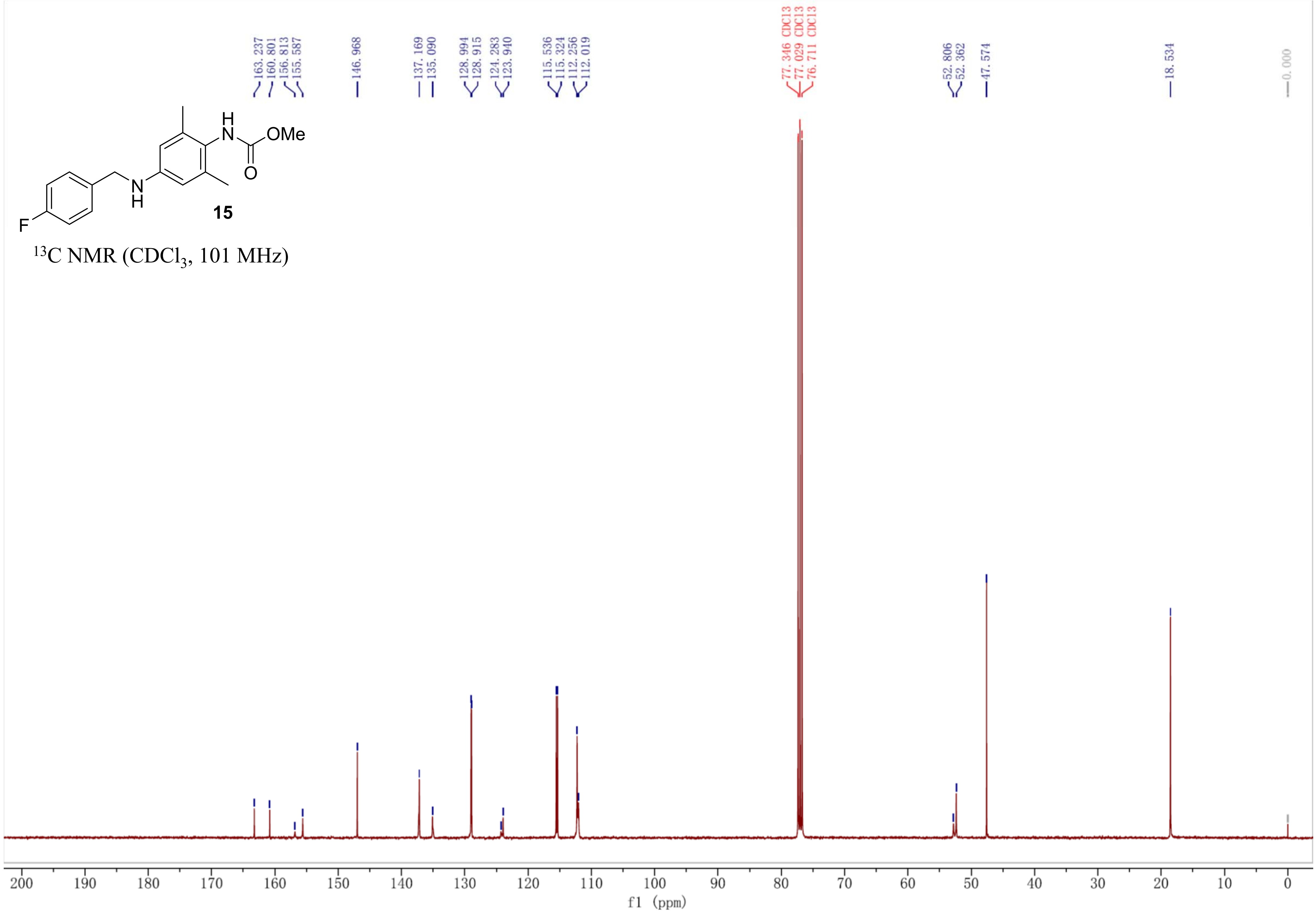

163.237
160.801
156.813
155.587
146.968
137.169
135.090
128.994
128.915
124.283
123.940
115.536
115.324
112.256
112.019
77.346 CDCl3
77.029 CDCl3
76.711 CDCl3
52.806
52.362
47.574
18.534
0.000
H
N
OMe
O
N
H
F
15
^{13}C NMR (CDCl$_3$, 101 MHz)
200 190 180 170 160 150 140 130 120 110 100 90 80 70 60 50 40 30 20 10 0
f1 (ppm)

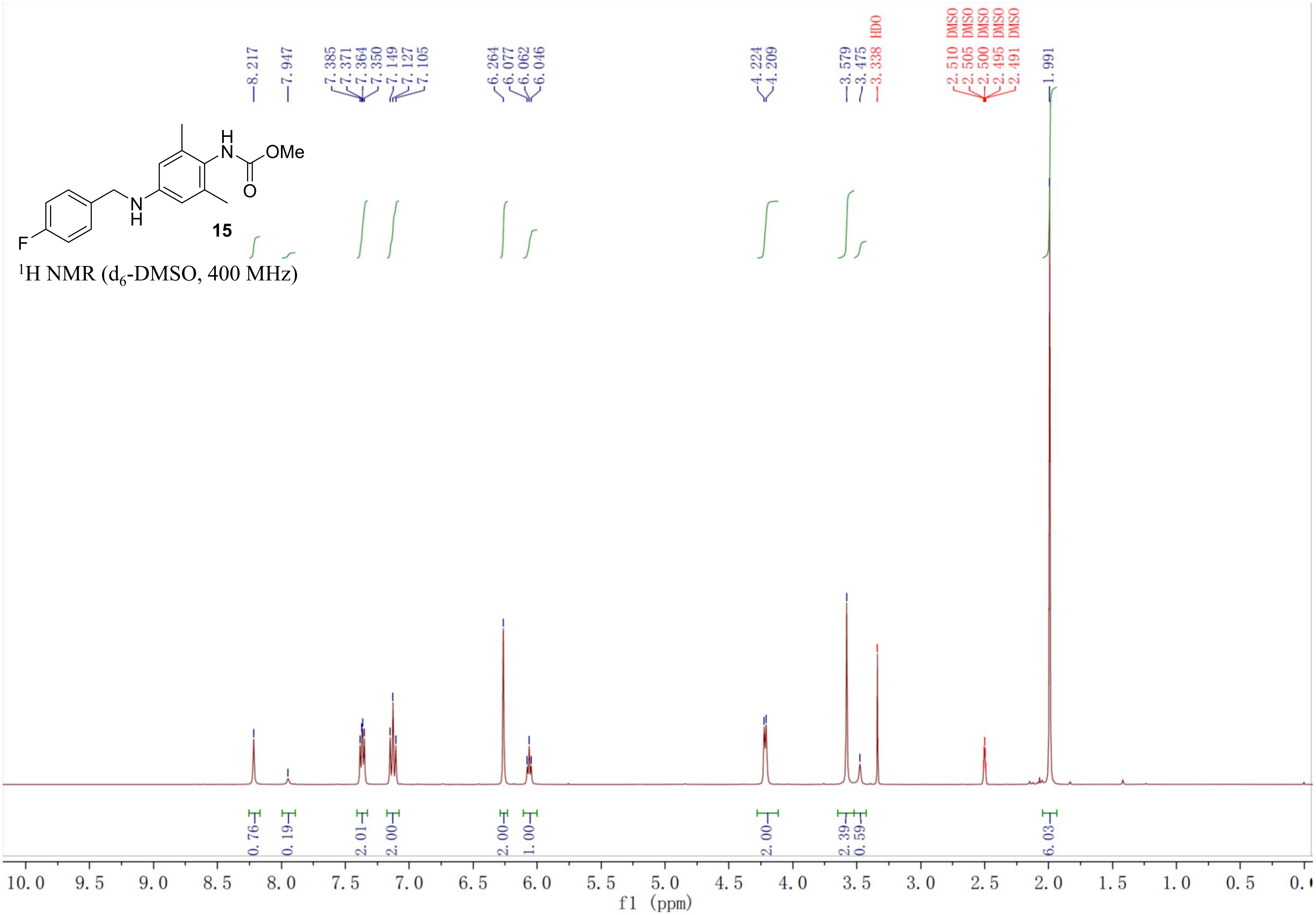

8.217
7.947
7.385
7.371
7.364
7.350
7.149
7.127
7.105
6.264
6.077
6.062
6.046
4.224
4.209
3.579
3.475
3.338 HDO
2.510 DMSO
2.505 DMSO
2.500 DMSO
2.495 DMSO
2.491 DMSO
1.991
H
N
OMe
O
F
N
H
15
1H NMR (d6-DMSO, 400 MHz)
0.76
0.19
2.01
2.00
2.00
1.00
2.00
2.39
0.59
6.03
10.0
9.5
9.0
8.5
8.0
7.5
7.0
6.5
6.0
5.5
5.0
4.5
4.0
3.5
3.0
2.5
2.0
1.5
1.0
0.5
f1 (ppm)

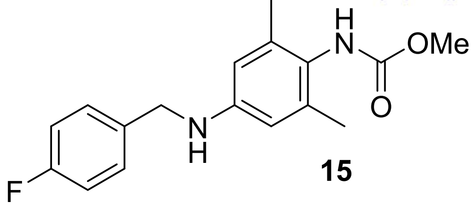

15

^{13}C NMR (d_6-DMSO, 101 MHz)

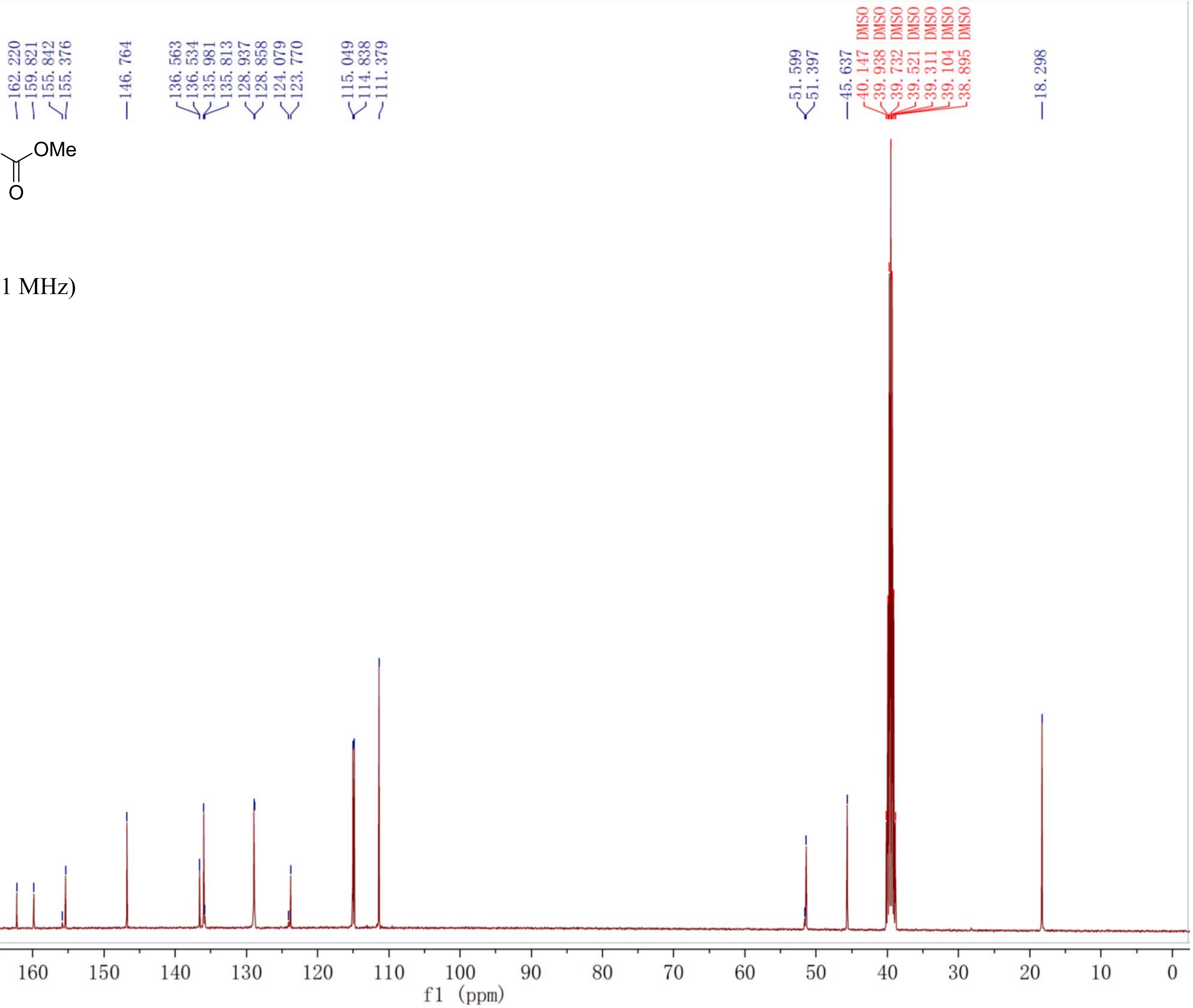

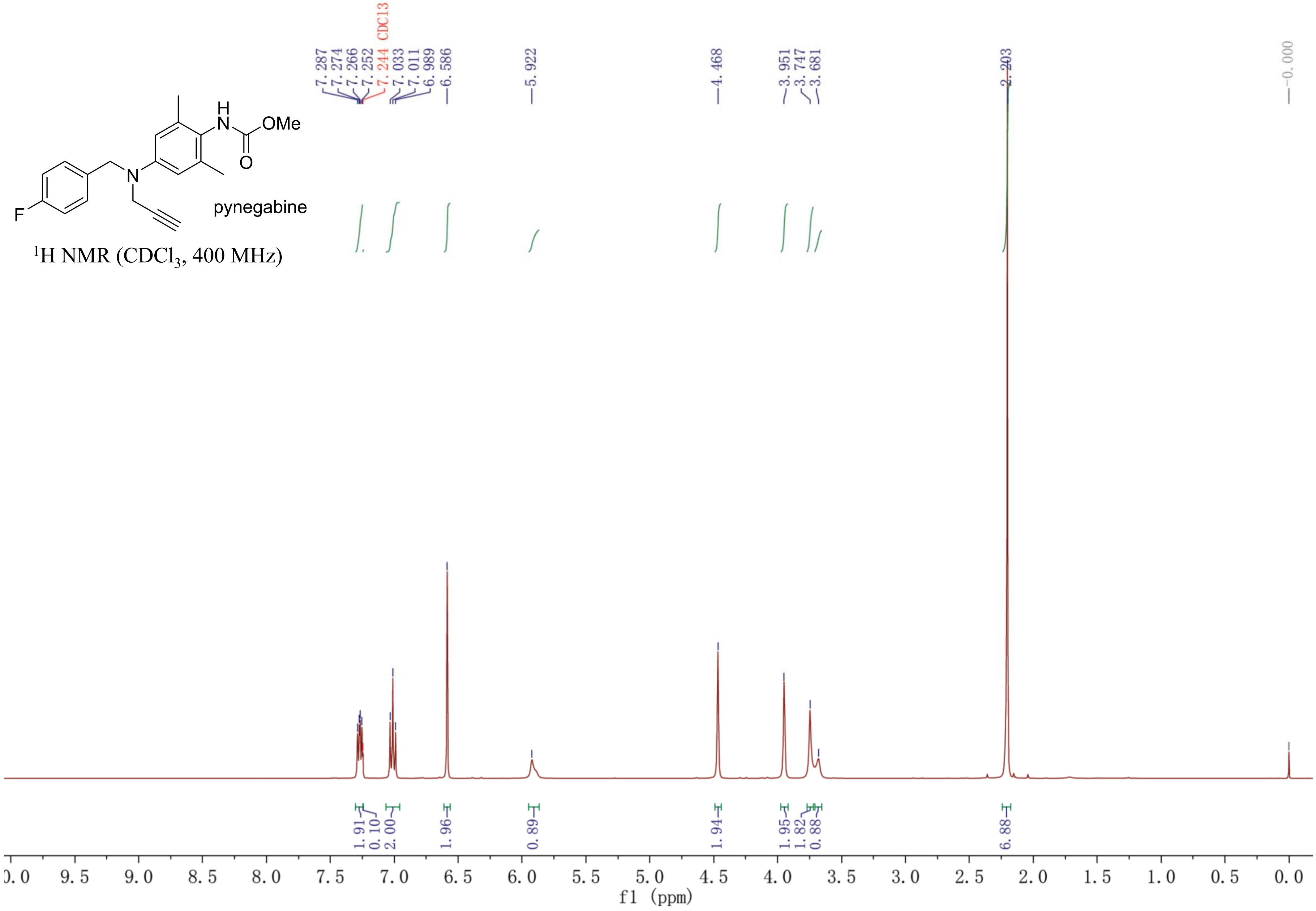
OMe
pynegabine
^{1}H NMR ($CDCl_3$, 400 MHz)
7.287
7.274
7.266
7.252
7.244 CDCl3
7.033
7.011
6.989
6.586
5.922
4.468
3.951
3.747
3.681
2.203
0.000
1.91
0.10
2.00
1.96
0.89
1.94
1.95
1.82
0.88
6.88
f1 (ppm)

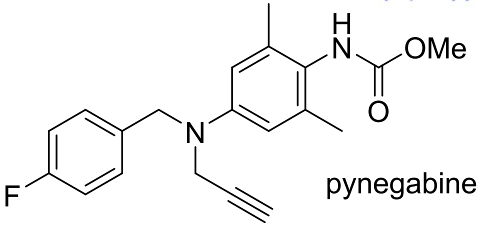

pynegabine

^{13}C NMR ($CDCl_3$, 101 MHz)

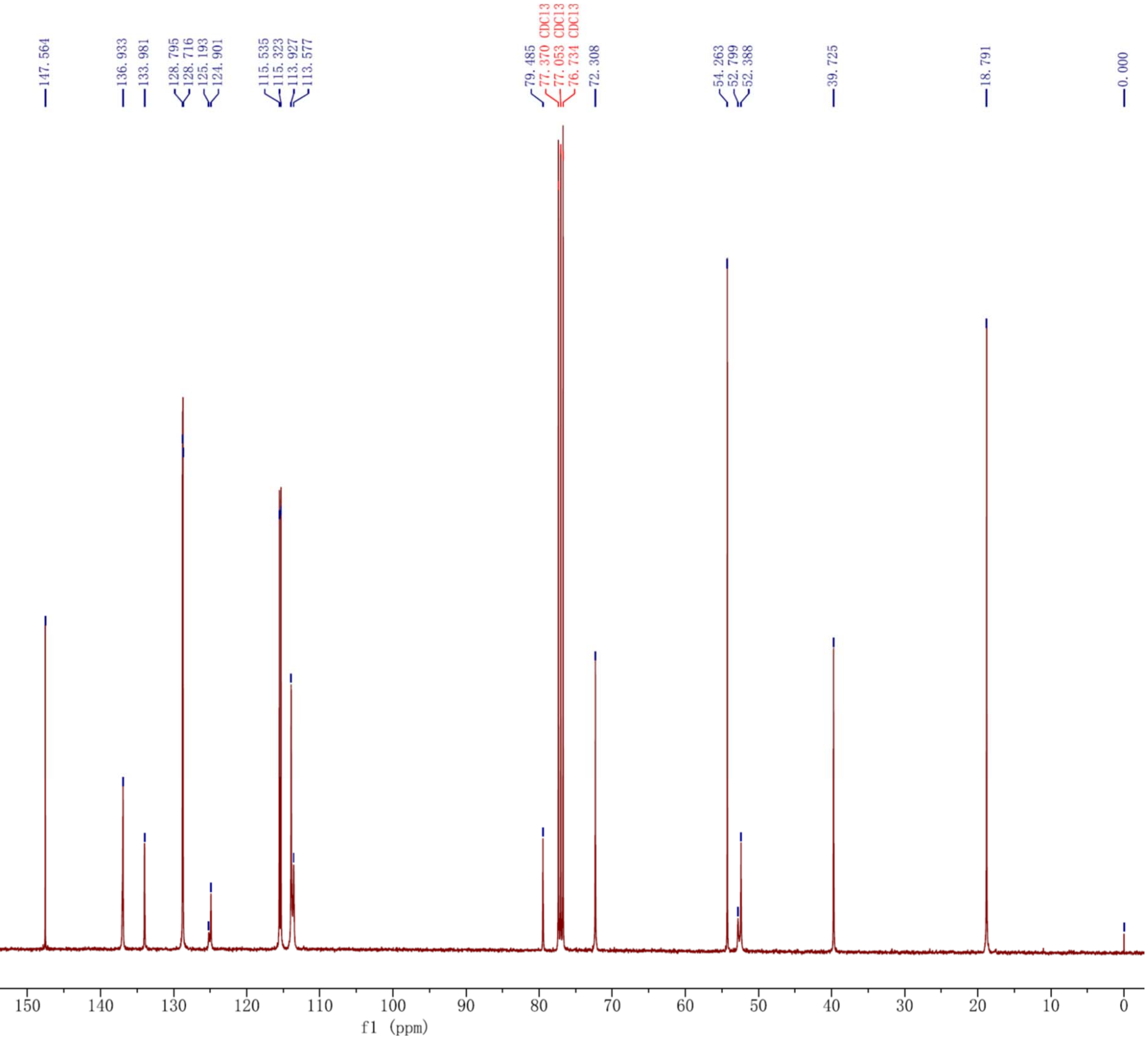

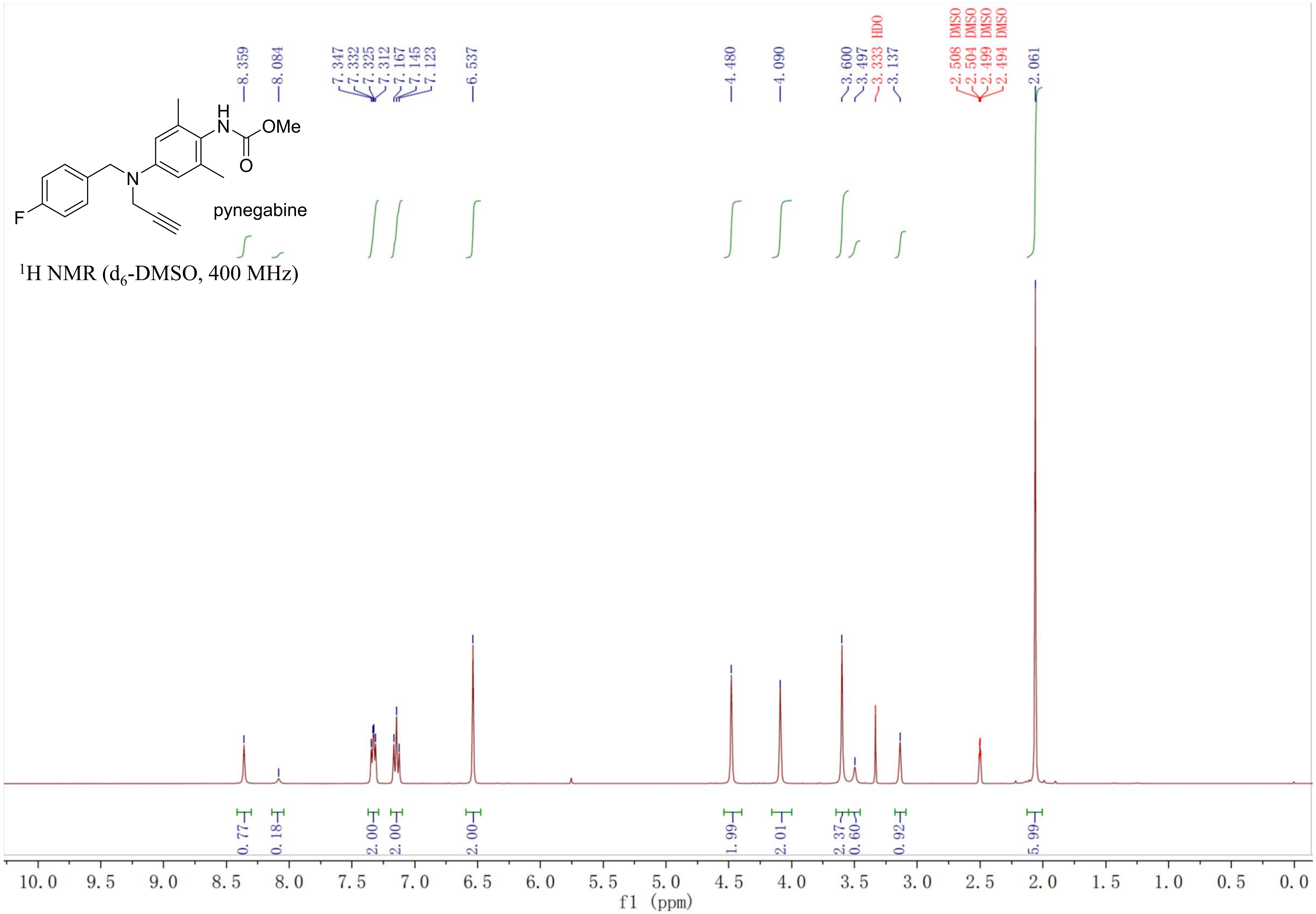

pynegabine
^{1}H NMR (d_6-DMSO, 400 MHz)
8.359
8.084
7.347
7.332
7.325
7.312
7.167
7.145
7.123
6.537
4.480
4.090
3.600
3.497
3.333 HDO
3.137
2.508 DMSO
2.504 DMSO
2.499 DMSO
2.494 DMSO
2.061
0.77
0.18
2.00
2.00
2.00
1.99
2.01
2.37
0.60
0.92
5.99
f1 (ppm)

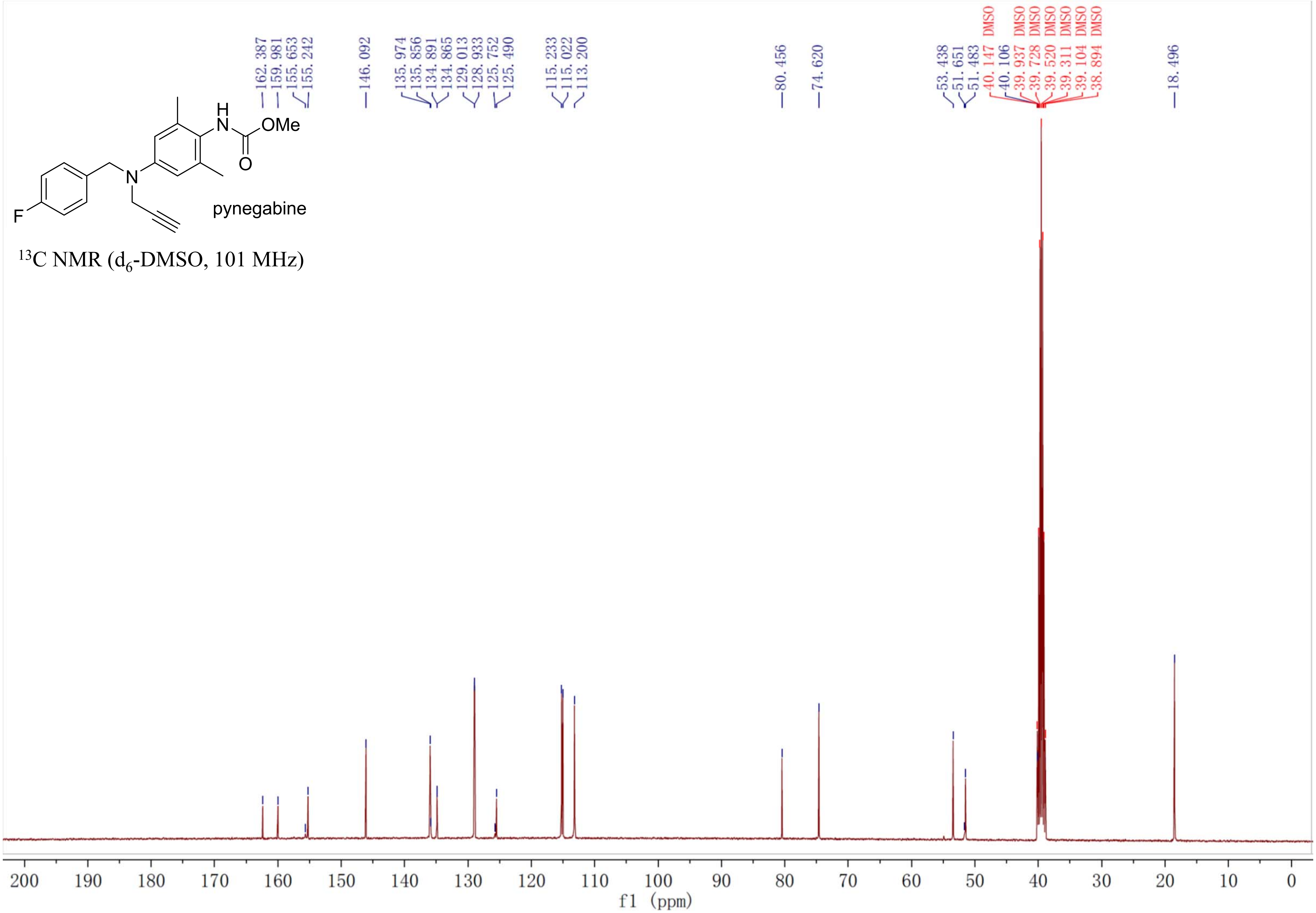

^{13}C NMR (d_6-DMSO, 101 MHz)

Elemental Analysis Report

Information				
Item	Organic Elemental Analysis			
Order No.	2306079545			
Number of samples	1			
Detection Mode	CHNS/O			
Date	2023.6.11			
Instrument	Elementar UNICUBE			
Sample	N（%）	C（%）	H（%）	S（%）
1	8.43	69.612	6.094	0
1	8.404	69.613	6.079	0
